# Occupational Health and Safety in the Least Developed Countries - A Simple Case of Neglect

**DOI:** 10.2188/jea.11.74

**Published:** 2007-11-30

**Authors:** M. Rabiul Ahasan, Timo Partanen

**Affiliations:** 1 Work Science Laboratory; Oulu University, FIN-90570 Oulu, Finland.; 2 Department of Epidemiology and Biostatistics, Finnish Institute of Occupational Health, Helsinki.

**Keywords:** work culture, social environment, industrial hygiene, occupational safety, obstacles

## Abstract

In many of the least developed countries, working people are significantly exposed to a number of occupational problems that may result in a deterioration of their health, safety and well being. These work-related problems are untenable, not only because of the occupational problems itself but also because of the simultaneous exposure to heat, dusts, noise, organo-chemicals, and biological and environmental pollution. This situation has existed for a long time due to various socio economic,geographical, cultural and local factors. The deteriorating situation of health and safety in the workplace may perhaps exist due to the inadequate resource facilities, economic constraints and lack of opportunity to conduct research and studies on the assessment of exposure-diseases associations. Officials, who are employed by the state, are not able to implement work regulations and labour legislation easily. Generally, they are not professionally trained and expert in the occupational health, industrial hygiene and/or safety fields, and thus, successful application and implementation of control measures are lacking. Steps to control work exposure limits have been ineffective, since national policies have been rare, owing to the multiple obstacles in preventing occupational problems. However, the major focus is on practical solutions to differing workers’ needs, consideration of which is very important, depending on the what the industrial entrepreneurs could reasonably to be expected to afford. Why there is a lack of motivation and effort regarding the development of health and safety-this paper explores some important issues, aiming to focus public attention on the legacy of national and international efforts. Examples are likewise given to show the real situation of health and safety in the least developed countries.

## INTRODUCTION

The least developed countries (LDCs) have been making great strides with regard to both industrialisation and economic reform. The rapid growih of industrialization and global market opportunity have therefore called for the implementation of occupational health and safety (OHS) as one of most important (i.e., Labour wellfare) issues. It focuses the ways in which the workers should be protected from various types of work exposures. OHS is defined as protection of workers from work-related disorders and/or exposure to hazards resulting from an unhealthy environment, and the incorrect way of accomplishing work-tasks (non-ergonomic posture, for instance). Generally, the concept of OHS has been expanded more to cover the prevention of work-related accidents and diseases, aiming for the recognition, evaluation and control of those special problems^1^^)^, together with the general living environment of the workers.

OHS is one of the basic rights related to many of the essential ingredients of workers’ wellbeing that are being denied in LDCs^2^^)^. Hundreds of millions of people in these regions are employed in conditions that deprive them dignity and value^3^^)^. The workers in many African countries, for instance, are the victims of work-related diseases and illnesses^4^^-^^11^^)^. OHS is yet to be realised fully, as it is an inevitable requirement of the workers in many factories and enterprises in Sub-Saharan Africa^12^^-^^13^^)^. Actual development of OHS in this region has a long way to go^30^^-^^31^^)^. Manual workers in many African nations^1^^-^^4^^)^ are involved with heavy work-tasks. The strenuous activities in hot working conditions are often presumed, which may inherently interweave with their poor health, unsafe acts and conditions.

Industrial medicine, work-safety, occupational health or industrial hygiene is not a familiar concept in many LDCs^12^^, ^^14^^)^. Local government initiative is poor in introducing intervention measures to improve health, safety and well-being. General health and safety protection is far from the reality, which may perhaps be true due to inadequate research and walk-through investigation on the assessment of exposure-diseases associations. The number of workers exposed to hazardous work-environment in their work place may be much higher than expected, because many of them do not report actual problems.

Specifying the responsibilities of all the parties concerned, a clear and achievable policy on “occupational health” and “workplace safety” is often lacking since illiteracy,poor social status, and poor economy erode their life style more than a pitiful work environment. Industrial production is affecting workers in many other parts of the LDCs due to lack of health and safety in the workplace^2^^)^. Musculoskeletal diseases are prevalent among the Chinese assembly line workers^15^^)^. Industrial workers are still living in the urban slum area in an unhygienic condition in India^16^^-^^17^^)^ and Bangladesh^18^^-^^19^^)^. Physical work environments are not the main areas of focus in Pakistan^20^^-^^21^^)^, Sri Lanka^22^^)^ and Nepal^23^^)^. There are also cases of acute negligence of health and safety in China^24^^)^, Vietnam^25^^)^, Thailand^26^^)^ and Korea^27^^)^. Further, due to low nutrition and concomitant infections or tropical diseases^1^^, ^^28^^)^, the workers in many developing nations are not able to perform their actual duties and work-performance. Integration of health and safety measures has not been widely recognized or implemented in some Latin American nations^29^^)^.

The industrial workers in LDCs are at a greater risk due to low level of production technology, limited capital resources, and lack of local and international supports. Indeed, individuals’ occupations are the economic source to support their families and relatives in many of these nations. Studies^32^^-^^33^^)^ showed that physically demanding work is occupationally stressful and unhealthy. Individual differences in physical capacity, age and nutrition can also influence job demand compatibility. Organisational and psychosocial factors are also related with OHS^34^^-^^35^^)^ because it may rise to a change in workers’ cognition (i.e., mental turmoil) and safety behaviour, which keep them hygienic and healthy. While quantitative demands increase, OHS become impoverished both physically and mentally^23^^)^. If certain measures are not maintained, the prevalence of occupational problems also contributes to physiological^36^^)^ and psychosocial reaction^34^^)^. These symptoms are comparatively higher in low productive work, while working in unhygienic environment^33^^)^. In addition, low quality of work content and lack of opportunities for competence developments are the common features of unsafe behaviour and poor health^26^^-^^27^^)^. Physically demanding jobs in a hazardous workplace with climatic stress and environmental pollution have been associated with unsafe behaviour at work. And thus, occupational illness is widespread in LDCs^37^^-^^38^^)^. Because they do have poor accesses to job training and education, and thus workers are exposed to various work-related problems. Workers should be educated and trained on the concept of work-safety, industrial medicine and/or occupational health. They should receive the benefit of proper training and education so that other constraints are minimized. In this context, vocational education is very important^39^^-^^40^^)^ for the manual or daily labourers to learn proper work technique. They should follow ergonomic way of doing activities through regular job training.

Preventive measures such as annual medical check-ups, and health and safety surveillance^41^^)^ would be more important than increasing industrial production. An introduction of health and safety intervention is therefore not itself a solution; if certain changes and effective measures are not effected rationalized (according to local need). Unless strong ties and collaboration are maintained between the developed and developing countries, the commitments of WHO^42^^)^ will never be succeed. To achieve WHO’s commitment^42^^-^^43^^)^ on “Occupational Health for All”, industrial health, occupational hygiene and safety aspects should be integrated with primary health care and safety. In this context, international organisation [e.g., ILO, WHO, UNDP, UNEP], western aid and development agencies (e.g., NORAD, DANIDA, CIDA, USAID, FINNIDA) as well as NGOs (Oxfam, CARE, World Vision, and so on) should be called upon for an immediate action.

## WHY DO THESE OBSTACLES EXIST?

Tracing back to sporadic incidents to today’s work-related problems, occupational problems have long been a part of the history of many developing nations. Very few entrepreneurs maintain high-quality work standards, partly because of economic constraints, and flexible attitudes of the regulatory agencies, and poor implementation of labour legislation. Since occupational diseases and safety problems are non-reviewed in many of the LDCs^44^^-^^46^^)^, and hence regional offices of ILO, UNDP or WHO face many obstacles against the tremendous demand for the elimination of occupational problems in these regions.

A suitable portfolio on the effective health and safety policy for sustainable development is yet to be made in completion and/or progress in the provincial or national level. Occupational diseases, as a whole, also rank at the top of health problems, because the national coverage (budget, expert manpower, vehicle, laboratory facility, etc.) for environmental monitoring and health surveillance are often lacking. The economic downturn in Asia, Africa and other nations may limit sanctioning a sufficient budget for workplace improvement. It is thus obvious that the workplace monitoring and workers’ periodic medical examinations are not carried out autonomously and/or automatically.

Studies^47^^-^^48^^)^ showed that accident severity rates are higher than expected in some countries. It is suspected that hazardous processes and second-hand machines causes some problems which are being exported to many of these nations without prior consent of correct information (e.g., user’s specification) to the local people^49^^-^^50^^)^. Workers’ health, safety and production are thus aggravated in LDCs with a deterioration of their physical work performances^1^^-^^2^^)^. The workers also are discriminated against unhygienic conditions especially in the small-scale industry^4^^)^. The main reason is that work-related problems in these industries rise due to the detrimental effect of the nonrecognition of their work and health^51^^)^.

Until recently, OHS issues are not targeted in the national agendas of many LDCs^30^^)^. Yet, neither the local government authorities and international organisations have paid special attention to improve the existing situation due to economic constraints and other barriers. Periodic inspection has not been even set up in the majority of state-owned industries or big enterprises. The current hygienic limit values in some countries are lower than standard^44^^, ^^52^^)^, and many workers are therefore being exposed to chemical hazards, pesticides etc.^53^^-^^54^^)^. OHS facilities are rarely subsidised even in any of the state owned industries for worker’s well-being. However health, hygiene, safety and welfare facilities are the basic rights of industrial workers, because they contribute national economy.

Many of the workers in the small-scale industries continue to live in squalor with unrecognised illness. Workers are also the victims of critical attitude of employers or entrepreneurs. Small scale entrepreneurs or employers are usually seen greedy, and often they are reluctant to allow, or to make the needed changes. Most of the employers are also cynical and unwilling to change any existing system such as work-line, shift-system, layout, organizational management, etc. Their attitudes are such that any change is seen as a waste of time and resources, a hindrance to production, and loss of production.

The national safety and health policy that includes identification, evaluation, prevention and control of occupational hazards is yet to be developed. Work disability (by proper diagnosis of their health illness, etc.) in cases involving accidents and occupational diseases made by the occupational health officials and/or safety experts is not available. Because the concerned organization [e.g. Department of Labour, Department of Industry, Public Health Officials] established for OHS have no such facilities (budget, equipment, vehicles or personnel). Factory inspectors are not generally expert in the specialised area of industrial medicine or work-safety. Industrial hygienist, safety managers or occupational health specialists are rarely available. They are not in a situation to examine the relevant problems of OHS. In many cases, factory inspectors are too lax with regard to oversights by the employers, even when their own neglect contributes to the problems. Often, the officials are seen as ambitious for their own personal interests, not to the duties and responsibilities to which they are assigned. It takes months to acquire any work-related information, such as the types and number of occupational accidents and injuries. The work regulations and labour legislation are seldom practised especially in the small-scale industries or agro-based rural industries. Inspectors usually claim that visiting remote places are too difficult mainly because of communication problems. At the individual industries, consultation service from University level institutes or any research centres seems to be a costly luxury.

When safety and health efforts are based on work-related information, the management should be able to direct the activities by setting appropriate goals, and by planning organising and controlling the activities accordingly. While implementation of work-regulation and labour legislation is vital as the state intervention, international financiers such as the World Bank and International Monetary Fund put pressure on the local governments. Structural adjustment, for instance, may hamper an individual country’s public responsibility for the implementation of health and safety.

## OHS-IS THERE ANY MISUNDERSTANDING AMONG THE PROFESSIONALS?

OHS is an essential component of industrial development and social progress. OHS is also a growing social concern since production economy and environmental protections are related. It is the professional speciality concerned with the prevention and control of work-related problems. However, it is perhaps more interesting to examine how different professionals in LDCs use the term, ‘OHS’ according to each individual’s understanding. How do they conceive of its significance as associated with “work-related health” or “non-work-related safety”? OHS could have also different meanings from various directions such as ‘need’ ‘quality’ and ‘inequality’-those are ambiguous to each group of professionals. There could be an unintentional misunderstanding however since individuals working in different disciplines [e.g., ergonomists, medical doctors, hygienists, or safety inspectors] do not use of the same or similar terminology in different views of industrial health and occupational safety as a particular problem. Technical terms should therefore be established with meanings and understood by all the personnel (or officials) who are involved with industrial health and/or work safety. The technical approach usually treats as an objective fact, seeking to measure the magnitude of safety. In some countries, for instance, health professionals seek patient’s problems without looking at their occupational safety problems. OHS services therefore need to be comprised of a curative occupational medicine for the workers suffering from occupational diseases.

The practical concern of an ergonomists is to improve the design of workstations, and worker’s safe manipulation of the tools, equipment and machinery. Most industrial hygienists look for environmental issues related to human health and epidemics. Thus, it may be contested to the professionals as a meaning of their actual task of health and safety. A contemporary society may however respond to the “occupational health” and “safety issues” differently. It is also important to define the occupational tasks along with OHS for specific people. Therefore, measures taken to eliminate occupational problems are to be specified on various aspects for making a major contribution to a clear understanding of OHS. This is especially important for the development of guidelines, health and safety plans, drafting and revision of disease prevention and safety campaign.

To renew socio-cultural attitudes toward safety behaviour or healthy attitudes, we need to upgrade the curriculum of OHS, based on the area, class, labour politics and regional culture. Industrial hygiene is another emergency part of work environmental monitoring for which workers and entrepreneurs’ awareness should be enhanced. It would benefit them if, at least, awareness training would be launched for occupational safety and environmental health. To implement the full concept of OHS, professionals should emphasize good examples showing mobile safety shows, for instance. In the form of audio-visual pictures, safety posters, pamphlets, gimmicks, and/or safety comics, training courses should adopt effective measures. Correct information should be disseminated in the industrial education or job training session. In order to meet the challenges for industrial development, education and training in each of the LDCs on the promotion of health, assessment, supervision, monitoring are the other parameters supplementing of work regulations.

## SOME RECOMMENDATIONS FOR LOW-COST IMPROVEMENT OF OHS

Implementation of OHS-measures is now a global issue, which should be considered as a matter of urgent attention. For all the level of development, preventive programs and control measures should be effective for long term benefits. Each of the individual countries should contribute to the cost of such measures. Reichs and Okubo^55^^)^ emphasised for national and international strategies. Low-cost improvement seems to be effective through the participatory programs (workers, entrepreneurs and regulatory agencies) the collaborative efforts^56^^-^^62^^)^, which is the key element for the improvement of the workplace. Phoon^40^^)^ and Tomberg^63^^)^ found that ergonomics principles could be better solutions for minimizing work-related problems. However, ergonomics measures should correctly fit with the implication of indigenous characteristics of local workers and working conditions. Job training is of the utmost necessity for growing safety and health consciousness^64^^-^^65^^)^. In this regard, systematic workplace surveys are necessary for routine-wise planning of jobs and tasks. By enlarging the worker’s span of control over the existing situation, feedback from workers has to be achieved through the recognition and appreciation of health and safety. Together with many other factories, an in-plant services system could provide safety and health surveillance. Workers must be encouraged to report all types of occupational problems so that immediate action could be found for easier solutions. In order to educate people in the ways in which work-related problems are related, grass-root level vocational training programs should be launched. Improvements are to be made not only to increase production, but also step by step progress is necessary in the areas of industrial health and hygiene. OHS should be linked to the national health plan, as well as safety surveillance for non-ergonomic situations^41^^)^. The best way is to ensure the long-term sustainability of targeting important actions, such as to control work-related exposures Research and field studies^66^^)^ are needed with the collaboration with the developed countries^67^^)^ to better harmonise all aspects of work, health and safety. Workplace investigations should be carried out more often to monitor the work sites for anti-epidemic surveillance at various levels. Budgeting for collaborative researches and field studies should be included in annual budget planning. Policy makers and employers need to ensure that provision of a safe work environment is a key consideration in all investment and production decisions, and the factory workers are to be involved in those decisions. Factory inspectors should be innovative and excel in practice; otherwise prevention and control will be neglected due to other pressures in the economy. Strict policy should be adopted by the regulatory agencies to enforce work standards and labour legislation^68^^-^^70^^)^. Effective measures should be extensive to monitor interventions, following up the early signs of chronic overload, exhaustion and injury. In order to prevent occupational problems, the concerned personnel should be devoted to their official duties for the implementation of work standards and labour legislation. There is a need of honest commitment to stimulating the control of work-related problems. Regulations should periodically be implemented on a case by case basis. The existing regulations should be further enforced rather strictly than trying to eliminate all problems at once. By the initiative of the management of the regulatory organization work injuries may be reduced substantially. A comprehensive policy is to be adapted for the legislative containment^68^^-^^70^^)^. OHS should be strengthened so that every nation can meet the need for promotion of heath and safety. A high priority option must be placed on the national agenda. International collaboration is to be called for in order to meet these challenges in reality.

## DISCUSSION

With the opportunity of the global free marketplace, LDCs are advancing with rapidly growing industrial development. Workers in these nations also represent large proportions of the global work force however they receive lower levels of attention. In the elimination of work-related problems, national planning and programs for the strategy of launching OHS measures is lacking. There can be no solution if traditional work practices are not changed to better practice and/or altering the traditional methods. To improve health, safety and well being, yet, very little has been an adopted by the government and international organizations. There is always a lack of funds and other problems for launching preventive programs, especially in industrial safety and occupational health. These are however susceptible to control by means of timely prevention. These measures should be taken into consideration by an appropriate step from the grass-root level. In this regard, LDCs needs attention from the international communities to improve the existing situation.

## CONCLUSION

Despite the global commitment from the international organization, why implementation of proper measures are not easily put into place, the critics, comments and examples came from the authors’ experiences, while working in a developing country and surveying various industrial workplaces. A series of questions is however to be confined to such issues. As such, how can we define the main tasks of factory inspectors and regional centers? How can we assess individual’s responsibility for correct supervision and inspection ? Who is going to measure and evaluate essential parameters of occupational health and work-safety? Who will follow up workers’ health, and treatment of occupational diseases and illness? Which recommendation will be correct for each of the individual nations for the control, prevention and the management of work exposures? How will the universal slogan of OHS attract more public attention for workers’ awareness?
